# Indents

**Published:** 1918-02

**Authors:** 


### INDENTS

☞ **Brief Articles of Technical or Scientific Value will receive notice and comment. Contributions invited.**

*Hypodermic Needles*

All needles employed for conductive anesthesia should be made of iridio-platinum as steel needles break so easily and the broken part is very hard to locate and remove. The iridio-platinum needle can be sterilized by heat and will not rust and break. It is so easily sterilized as to reduce to a minimum the danger of infection. Needles made of nickel may also be used safely.

*To An Old Tooth*

Farewell, old tooth, thy parting gave me pain,
And yet, forsooth, I would not have thee back again.
To abide with me.
And as I gaze upon thy wornout form, how innocent thou doth appear,
One would not think the howling storm that filled my head with pain and fear,
All came from thee.

—R. M. Weber, in *American Dentist.*

*To Prevent Modeling Compound from Sticking to Fingers*

So many of us use modeling compound for minor operations, heated over a dry heat, but we find that it sticks to our fingers and hands and sometimes causes a blister.

To prevent this, if the hands are subject to being dry, moisten them a little, then dry; if not, just sprinkle a few sprays of talcum powder over the palm of each hand, rub it in good, then go ahead and shape the compound into any shape you wish without any fear of it sticking to your fingers or hands. If you are preparing the compound to use in the mouth, the odor of it is off-set by the sweet perfume of the powder.

Moistening the hands only helps to keep the powder on them longer. The small amount of the powder that is rubbed into the compound does not affect it in any way. —J. V. Chandler, in *Digest*.

*Sterilizing the Operative Field* A stable solution for use in sterilizing the operative field is made by mixing menthol, one gramme, with iodine, one gramme, and benzol, 25 c.c. Just before making an injection for conductive anesthesia, dry the operative field and apply the solution. It not only sterilizes the field, but produces sufficient obtunding of sensitiveness to make the insertion of the needle less painful. It leaves no detrimental effect on the mucous membrane as does iodine tincture.—J. E. Nyman, in *Dental Review*.

*Forysth and the Halifax Disaster* Immediately following word of the terrible disaster to the city of Halifax, a special unit was organized consisting of members of the visiting staff of the Forsyth Dental Infirmary, whose intended duty was for treating those suffering from broken jaws. The offer of the services of this unit was made to Gov. McCall, and to the chairman of the Committee on Public Safety, Mr. Endicott.

This unit is very complete and comprises one of the most skillful general surgeons in the city, three dental operators with special training and experience in the treatment of the jaw, a number of dentists skilled in the construction of the particular appliances necessary for this work, graduate dental nurses, a mechanical laboratory man of experience, together with all of the supplies, appliances, bandages and laboratory apparatus so that the unit will be able, if necessary, to establish its headquarters in one of the cars and operate absolutely independent of any local aid.—Harold DeW. Cross, Director.

*A Perfect Alibi* Ralph's father is a doctor, and Ralph naturally likes to play he is of the same profession, using an old hat and an old medicine case of his father's to dress the part properly. One day the telephone rang, whereupon Ralph called out: "Somebody wants me!" and caught up his hat and case and hurried out.

"Come back and shut the screen door, Ralph," called his mother, and insisted until he obeyed.

Later, when he came in, looking very sober, she queried sociably: "Well, Dr. Ralph, how did you find your patient?"

"Dead!" he replied, accusingly. "He died while I came back to shut the door."

*Sterilizing Hand Brushes* If you have not used it, I would recommend a solution of potassium bichromate for keeping the hand brush antiseptic. After you have used the brush, it should be washed out, put in the sterilizer and boiled, and when it is taken out of the sterilizer it should be placed in a glass container filled with the bichromate solution. After the container is thoroughly cleaned and filled with boiled water taken from the sterilizer, then put in just enough bichromate to give the water a deep orange color. It is not necessary to measure the exact percentage, because a little more than is necessary does no harm. I prefer it to lysol. It is a good antiseptic, and is not harsh on the hands. It is a good deal cheaper, too, costing about ten cents for four ounces. It is very comfortable to use on the hands, and does not leave any disagreeable odor. Brushes kept in this solution will remain sterile until you are ready to use them. With regard to handpieces, we put our handpieces right in the autoclave and sterilize them.—Dr. Ash, in *Cosmos.*

